# Prediction of 123I-FP-CIT SPECT Results from First Acquired Projections Using Artificial Intelligence

**DOI:** 10.3390/diagnostics15111407

**Published:** 2025-05-31

**Authors:** Wadi’ Othmani, Arthur Coste, Dimitri Papathanassiou, David Morland

**Affiliations:** 1 Médecine Nucléaire, Institut Godinot, 51100 Reims, France; wadi.othmani@reims.unicancer.fr (W.O.); acoste@chu-reims.fr (A.C.);; 2 CReSTIC, UR 3804, Université de Reims Champagne-Ardenne, 51100 Reims, France; 3 Laboratoire de Biophysique, UFR de Médecine, Université de Reims Champagne-Ardenne, 51100 Reims, France

**Keywords:** Parkinsonian disorders, dopamine plasma membrane transport proteins, tomography, emission-computed, single-photon, basal ganglia, iodine-123, artificial intelligence, neural networks, computer

## Abstract

**Background/Objectives:** 123I-FP-CIT dopamine transporter imaging is commonly used for the diagnosis of Parkinsonian syndromes in patients whose clinical presentation is atypical. Prolonged immobility, which can be difficult to maintain in this population, is required to perform SPECT acquisition. In this study we aimed to develop a Convolutional Neural Network (CNN) able to predict the outcome of the full examination based on the first acquired projection, and reliably detect normal patients. **Methods:** All 123I-FP-CIT SPECT performed in our center between June 2017 and February 2024 were included and split between a training and a validation set (70%/30%). An additional 100 SPECT were used as an independent test set. Examinations were labeled by two independent physicians. A VGG16-like CNN model was trained to assess the probability of examination abnormality from the first acquired projection (anterior and posterior view at 0°), taking age into consideration. A threshold maximizing sensitivity while maintaining good diagnostic accuracy was then determined. The model was validated in the independent testing set. Saliency maps were generated to visualize the most impactful areas in the classification. **Results:** A total of 982 123I-FP-CIT SPECT were retrieved and labelled (training set: 618; validation set: 264; independent testing set: 100). The trained model achieved a sensibility of 98.0% and a negative predictive value of 96.3% (one false negative) while maintaining an accuracy of 75.0%. The saliency maps confirmed that the regions with the greatest impact on the final classification corresponded to clinically relevant areas (basal ganglia and background noise). **Conclusions:** Our results suggest that this trained CNN could be used to exclude presynaptic dopaminergic loss with high reliability from the first acquired projection. It could be particularly useful in patients with compliance issues. Confirmation with images from other centers will be necessary.

## 1. Introduction

### 1.1. Clinical Context

The diagnosis of Parkinsonian syndromes usually relies on clinical evaluation. However, even after a thorough examination, the etiology remains ambiguous in up to 30% of the cases, requiring further investigation [1]. While Magnetic Resonance Imaging (MRI) provides detailed anatomical information, it lacks the functional imaging capabilities necessary to assess presynaptic dopaminergic function directly. Cerebral Single-Photon Emission Computed Tomography (SPECT) with Dopamine Transporter ligands (DaT), such as 123I-FP-CIT (123I-N-ω-FluoroPropyl-2-β-Carboxymethoxy-3β(4-Iodophenyl)norTropane) or ioflupane, is a valuable tool to detect or exclude a striatal presynaptic dopaminergic deficit in these scenarios [2]. It allows for the visualization and quantification of dopamine transporter availability, making it uniquely suited for detecting early dopaminergic deficits characteristic of Parkinsonian syndromes. Although other functional imaging modalities like 18F-DOPA PET exist, they are less widely available and more costly, limiting their routine clinical use. DaT-SPECT has been shown to alter diagnoses in up to 51% of patients and change treatment plans in up to 49% [1,3]. Because of this high diagnostic yield, guidelines published by the European Association of Nuclear Medicine (EANM) and the Society of Nuclear Medicine and Molecular Imaging (SNMMI) recommend DaT-SPECT in cases of clinically uncertain parkinsonism [4]. Despite its benefits, the SPECT acquisition process presents practical challenges, such as the requirement for patients to keep still during the acquisition, at least 30 min, which is essential to obtain the necessary projections for accurate reconstruction and diagnosis [4]. In the literature, it has been reported that 3 to 4% of DaT-SPECT scans were inconclusive due to patient movement during acquisition [3,5,6]. Reducing the dependency on lengthy acquisitions by extracting diagnostic information earlier and from fewer projections could therefore be valuable.

### 1.2. Artificial Intelligence in Nuclear Imaging

Developing a Convolutional Neural Network (CNN) model to predict examination results from the first acquired projections may address this issue. A CNN is a specialized type of deep learning algorithm within the broader field of artificial intelligence (AI), designed to process and analyze visual data by automatically detecting patterns and features. These networks consist of multiple layers that can learn to identify and extract relevant features from images, making them effective for image classification tasks. Prior studies demonstrated robust results in accuracy, sensitivity, and specificity using neural networks on full SPECT images, but these methods required all the tomoscintigraphy projections and reconstructions, meaning they still depend on a full-length acquisition (Table 1). In nuclear medicine, AI applications have focused on image reconstruction, enhancement, and diagnostic classification [7].

### 1.3. Objective

This study aimed to develop a CNN model able to assess non-pathological DaT-SPECTs with a high sensitivity, using only the first projection (anterior and posterior at 0°) acquired in 30 s, instead of 30 min. By accurately identifying normal scans using minimal input data, such a model could enable clinicians to confidently shorten the acquisition for patients who are unlikely to have dopaminergic deficits. Additionally, our secondary goal was to build saliency maps which highlight the most relevant areas of the images in the classification process, in order to analyze the logic underneath [17].

## 2. Materials and Methods

### 2.1. Data Collection and Labeling

A total of 982 DaT-SPECT acquisitions performed in routine practice at our institute from June 2017 to February 2024 were retrospectively collected. Inconclusive DaT-SPECT due to movement artifacts during the acquisition were not included to ensure accurate model training, which was the only exclusion criterion. All imaging data were acquired using a Symbia Intevo 6 SPECT-CT system (Siemens Healthineers, Erlangen, Germany), including a dual-head SPECT camera equipped with 5/8″ crystals and fan-beam collimators, as they improve count sensitivity and spatial resolution compared to parallel-hole collimators [18]. Each acquisition included 120 projections (128 × 128 matrix), with each step (comprising two projections) lasting 30 s over a 360° circular orbit, performed at least 3 h after the administration of 90 MBq of 123I-FP-CIT. DICOM files of the SPECT images were retrieved and anonymized, then the first 2 native projections (anterior and posterior at 0°) were used for the CNN without any reconstruction algorithm.

Each examination was initially analyzed by a senior physician on a dedicated interpretation console (ESoft, Siemens). Two independent physicians reviewed and labeled each DaT-SPECT as follows:

- 0: No loss of dopaminergic activity
- 1: Doubtful exam but towards no loss of dopaminergic activity
- 2: Doubtful exam but towards a loss of dopaminergic activity
- 3: Loss of dopaminergic activity

Discrepancies were resolved by consensus.

**Figure 1:**
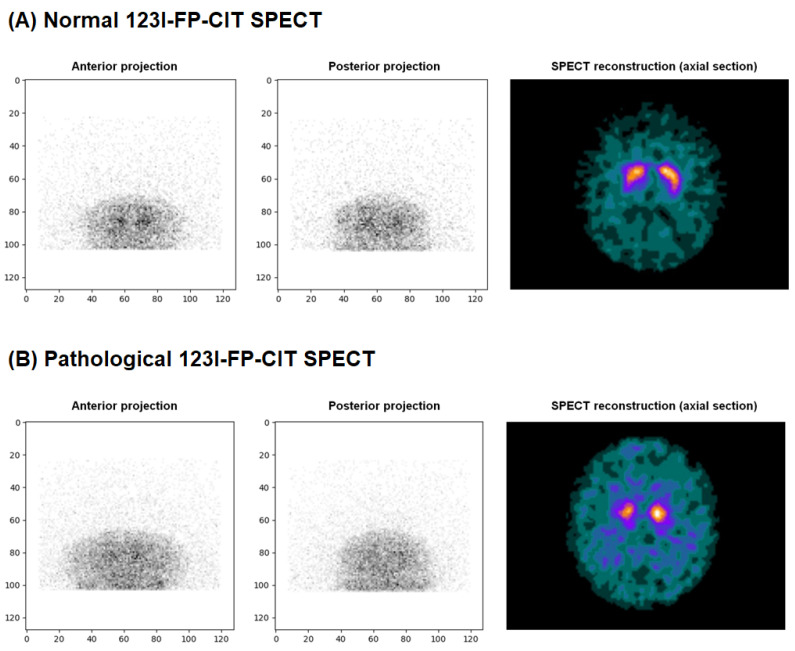
Examples of a normal and a pathological DaT-SPECT from the training set, displaying the anterior and posterior projections, along with an axial section of the corresponding full SPECT reconstructed images used for interpretation. (**A**) In the normal case, the striatum exhibit a well-defined ‘coma’ shaped uptake. (**B**) The pathological example shows markedly reduced striatal uptake and increased relative background activity, indicative of presynaptic dopaminergic deficit.

Since the goal of the model was to avoid any false negatives, DaT-SPECT labeled as 0 were considered ‘negative DaT-SPECT: normal’, and those labeled as 1, 2, or 3 were considered ‘positive DaT-SPECT: abnormal’ to maximize sensitivity and confidently exclude presynaptic dopaminergic loss (examples in Figure 1). This conservative binarization ensured that even doubtful scans (scores 1 or 2) were treated as abnormal in training, effectively instructing the model in being cautious for anything not clearly normal. The rationale is that a false-negative (missing an abnormal case) would be clinically unacceptable, while false-positives (flagging a normal case as abnormal after 30 s of acquisition) could be tolerated given that the standard full SPECT would catch the error.

An independent test set of 100 DaT-SPECT exams (50 normal, 50 abnormal) was set aside a priori before model training; this test set was not used at all in model development, serving only for final performance evaluation. The final labeled dataset was split into training and validation subsets (70% and 30%, respectively) using randomized stratification that preserved the same proportion of normal/abnormal cases in each subset.

### 2.2. CNN Model Development

The model was realized in adequation with the TRIPOD-AI guidelines checklist available in the appendix (Appendix A). The CNN was trained on a CPU AMD Ryzen 5 5500 U, 16.0 GB RAM. The following software packages were used: Python v3.10.9 on Jupyter Notebook (Anaconda environment, v23.7.4), Pytorch v2.3.0 for machine learning, Sklearn v1.4.1 for metrics calculation, and Matplotlib 3.8.3 for results visualization.

Preprocessing steps involved extracting the anterior and posterior projections at 0° from DICOM files, converting them into tensors, and normalizing them based on the maximum value of each image. According to the EANM/SNMMI practice guideline of 2020, data evaluation should consider age as relevant information [4,19], thus the age was extracted and each DaT-SPECT had a decade value attached for classification optimization.

**Figure 2:**
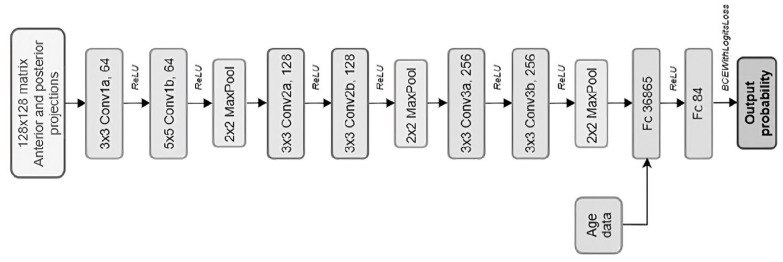
Convolutional Neural Network model used. Conv: Convolutional layer; MaxPool: max pooling layer; Fc: fully connected layer; ReLU: Rectified Linear Unit activation function.

The CNN architecture (Figure 2) is based on a Visual Geometry Group (VGG)-like structure, a standard deep learning model for 2D classification with multiple layers [20]. It was selected in pilot experiments aimed at reducing the CNN size without loss of performance, thereby minimizing inference costs and the risk of overfitting. For each DaT-SPECT, the input data were the first two projections and the age, then this architecture includes 3 blocks of 2 convolutional layers using Rectified Linear Unit (ReLU) as activation function, each followed by a ‘max pooling’ operation for downsampling and finally flattened by 2 fully connected layers while the age data is concatenated. We used 64, 128, and 256 filters in the first, second, and third conv blocks, respectively.

This model was compiled using the Adam optimizer with an initial learning rate of 0.0001, and the metric used to analyze performance was accuracy. The loss function employed was “Binary Cross Entropy With Logits Loss”, suitable for a two-class problem [21]. The model was trained over 60 epochs with a batch size of 6, which was a good compromise between training time and the risk of overfitting, as observed in pilot experiments [22].

Each epoch proceeds as follows:

(1) The model evaluates all DaT-SPECT in the training set, making predictions based on the current weights within the neural network.
(2) The discrepancy between these predictions and the actual status is measured by the loss function. The Adam optimizer then readjusts the neural weights within the network to reduce this discrepancy.
(3) Accuracy (i.e., the rate of correct predictions) is calculated using these adjusted parameters, completing this epoch.

The process then restarts from step 1 with the newly adjusted neural weights.

### 2.3. Threshold and Statistics

The output of the model was named “Pathological Confidence Index” (PCI), which represents the confidence of the model in the “abnormal” class prediction from 0 to 100%. As a binary classification, a PCI towards 0% represents a “normal” prediction, and towards 100% an “abnormal” prediction. Each prediction was compared with the corresponding reference status, and classified as True Positive (TP), True Negative (TN), False Positive (FP), or False Negative (FN).

By default, a threshold of 50% on this probability would classify cases into normal vs. abnormal (i.e., ≥50% = abnormal). However, our goal was to maximize sensitivity (true positive rate) even at the expense of false positives. Therefore, these parameters were studied during the validation phase using a receiver operating characteristic curve to characterize sensitivity (Se) and specificity (Sp) at optimal threshold (defined as the PCI threshold giving the highest Se while maintaining a Sp >50% in the validation set). This approach allows to detect normal examination with high selectivity, which is recommended for this kind of early screening [23]. The model evaluates the DaT-SPECT from the validation set to observe how it performs on data it has not encountered during training. This step ensures the model is not only effective on training data but can also generalize its predictions to new images. Also, selecting the optimal threshold based solely on the training set would have likely introduced an inherent overfitting bias, leading to an overly optimistic estimation of performance. Instead, we determined the threshold using the validation set.

The model was then used with this predefined threshold to predict the status of DaT-SPECTs in the independent testing set. From that, parameters such as sensitivity, specificity, and accuracy were calculated. Confidence intervals (CI) were determined using the Wilson score interval method.

### 2.4. Saliency Maps

A method derived from Grad-CAM was used to generate saliency maps which help in visualizing the most impactful areas in the classification process [24]. Since the Grad-CAM method was built for multichannel images (like red–green–blue), we used the two projections as inputs, without modifications. The last convolutional layer’s gradients with respect to the predicted class were used to compute a heatmap over the input image. The resulting saliency maps indicate which areas of the projection images contributed most strongly to the model output (positive or negative). We generated saliency maps for test cases to verify if the CNN was focusing on clinically relevant anatomy such as the striatal region, and analyzing these saliency maps may explain the logic behind the true and false predictions.

## 3. Results

### 3.1. Included Patients

The study included 982 DaT-SPECT, 517 men and 465 women, with a mean age of 70.8 years. The independent testing set consisted of 100 scans (50 ‘normal’ and 50 ‘abnormal’), selected initially and thus not used in training or validation. The remaining images were divided into a training set (*n* = 618) and a validation set (*n* = 264), following the recommendation to allocate at least two-thirds of the dataset for training if the total size is reasonable (*n*  ≥  100) [11]. There were 19 inconclusive DaT-SPECTs due to patient’s movement artifacts, which were preemptively not included to ensure an accurate model training, as usually done in machine learning [25]. DaT-SPECT’s distribution in each set is shown in Table 2.

### 3.2. Training and Validation Outcomes

**Figure 3:**
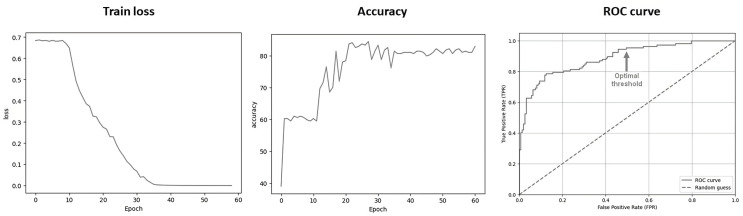
Evolution of training loss and accuracy (validation set) through epochs. ROC curve analysis in validation set. ROC = receiver operating characteristic. Optimal threshold = 1.8 × 10^−5^%.

After training, the loss (representing the difference between the predicted results and the actual results in the training set) and the accuracy (rate of correct predictions in the validation set) converged until the model’s performance showed an accuracy of 82.6%, without evidence of overfitting. The derived sensitivity and specificity were 79.4% and 84.5% in the validation set using the default threshold (PCI > 50%) (Figure 3). The optimal threshold (highest Se while Sp > 50%) was determined as a PCI > 1.8 × 10^−5^%, meaning all DaT-SPECT with a probability greater than 0.000018% for being abnormal were considered as abnormal. After applying the threshold, the model achieved a Se of 95,4% and a Sp of 50.6% in the validation set (Figure 3). This trade-off was deemed acceptable for our intended use-case of a rule-out screening.

### 3.3. Model Performances

The trained CNN model with the predefined threshold was evaluated on the independent testing set, consisting of 50 normal and 50 abnormal DaT-SPECT. The classification resulted in 49 TP, 1 FN, 26 TN, and 24 FP (Table 3). Performance metrics, with their 95% CI, were as follows:

- Sensitivity: 98.0% [89.5–99.6]; Specificity: 52.0% [38.5–65.2]
- Accuracy: 75.0% [65.7–82.5]
- Negative Predictive Value: 96.3% [81.7–99.3]
- Positive Predictive Value: 67.1% [55.7–76.8]

After adjusting for the 42% prevalence of abnormal DaT-SPECT in our full population using the Bayesian method, the unbiased PPV was 59.7% [53.2–67.0] while the unbiased NPV was 97.3% [88.5–99.7]. The trained model’s execution time for evaluating each DaT-SPECT, from extracting DICOM files to displaying the output, ranged between 0.03 and 0.06 s per DaT-SPECT.

### 3.4. Saliency Maps Analysis

**Figure 4:**
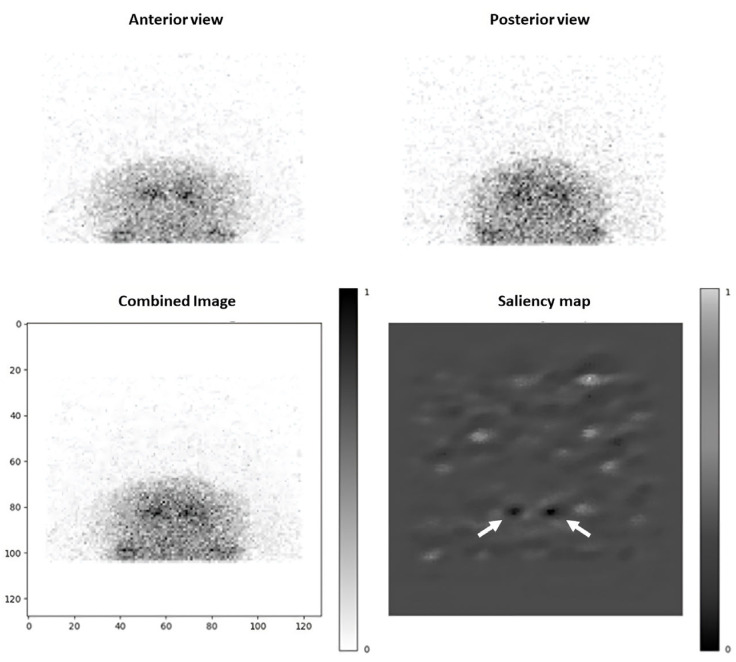
Example of a normal DaT-SPECT from testing set (age: 70 years), with first projections (top row), a mean combined image, and saliency map. Output probability of this examination to be pathological is estimated inferior to 10^−5^% (prediction: normal). The two dots pointed at by arrows represent basal ganglia area, and their black color illustrates these pixels’ gradient leaning toward a “normal” classification.

**Figure 5:**
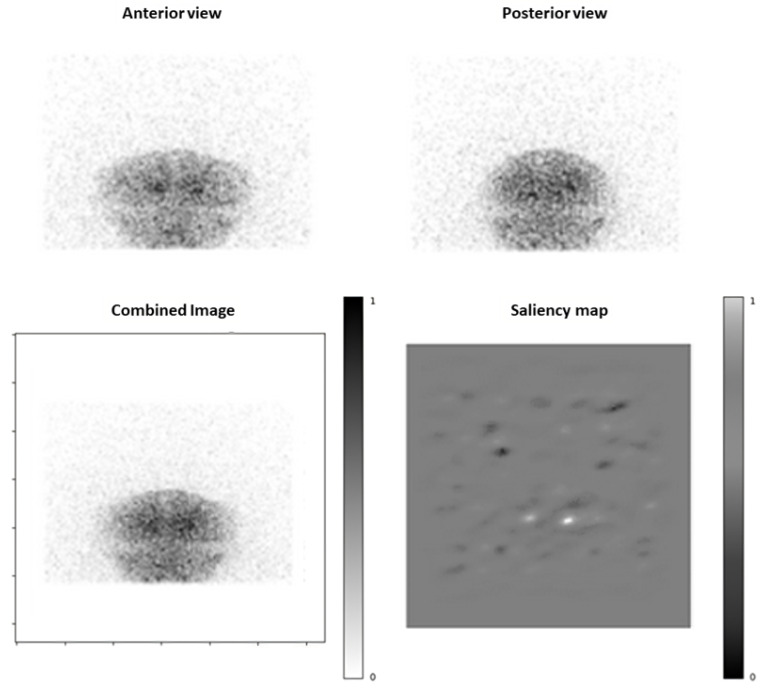
Example of a pathological DaT-SPECT from testing set (age: 80 years), with first projections (top row), a mean combined image, and saliency map. Output probability was predicted as abnormal. The two white dots in saliency map represent basal ganglia area, and their white color illustrates these pixels’ gradient leaning toward an “abnormal” classification.

The saliency maps generated provided insights in the areas of the images that contributed most significantly to the model’s classification. The most impactful regions corresponded to the basal ganglia, consistent with the presynaptic dopaminergic loss semiology (Figure 4 and Figure 5). Furthermore, the background noise also influenced the classification. We also observed some patterns of “full white” saliency maps, where all pixel gradients indicated an abnormal classification, which corresponded to DaT-SPECTs with a PCI of 100% This pattern could help in having a better specificity.

Regarding the only “false negative” (i.e., incorrectly predicted as normal) DaT-SPECT, we observed that even if the background noise was mostly leaning towards a “normal” prediction, resulting in a PCI of 2.1 × 10^−10^, the basal ganglia area showed a pathological pattern.

## 4. Discussion

DaT-SPECT acquisition takes a long time, which can be a problem in patients with Parkinson’s syndrome. Inconclusive DaT-SPECT examination due to patient movements represents about 4% of acquisitions. The aim of our model is not to replace SPECT acquisition, but to enable a certain number of patients (particularly those without dopaminergic deficits) to avoid a new acquisition in the event of the first not being compliant. A key strength of this model is the minimal preprocessing and processing time required, less than a tenth of a second per Dat-SPECT. In particular, cropping or mirroring were avoided, and only raw projections are needed.

The model demonstrated high negative predictive value, crucial for the reliable identification of normal DaT-SPECT. The specificity, intentionally reduced by adjusting the threshold, remained above 50%.

NPV is an extrinsic parameter that depends on the prevalence of dopaminergic loss among patients undergoing DaT-SPECT: the lower the prevalence, the higher the NPV [11]. However, in the testing set, the prevalence of abnormal DaT-SPECT was 50%, so higher than the 42% in the overall study population, which might underestimate the NPV.

Simple data augmentation methods like flips or rotations were avoided because the projections have fixed orientations and symmetry that carry diagnostic meaning (e.g., left-right differences). More complex augmentation strategies could have been used such as adding noise; however, in our pilot experiments, augmentations did not significantly improve sensitivity, and on the contrary, could mislead the model. Indeed, the signal-to-noise ratio is a critical information in the interpretation of DaT-SPECT in clinical routine, so we opted to train on the real projections, reflecting a real-world scenario and avoiding data generation biases [26,27,28].

Some additional clinical variables might have been added to enhance the neural network’s performance; for example, the sex of the patients. However, our pre-study analysis showed that including sex resulted in marginal changes in performance metrics, with sensitivity and specificity remaining comparable, thus we did not include them in the final model in order to optimize its computing time. Further tests might be interesting, especially with the newest deep learning architectures.

The saliency maps provided visual confirmation that the CNN focused on clinically relevant area to determine its answer. In most cases, the area of the basal ganglia was the main region influencing the decision. This area consistently mirrored the true status of DaT-SPECT (i.e., indicating pixel gradients leaning towards normal or abnormal accurately, even in cases of incorrect final predictions). This suggests a potential benefit in focusing on these areas rather than inputting the entire matrix into the CNN. Such an approach has the potential to enhance overall accuracy, even when using only two projections. This kind of interpretability is crucial for clinical adoption of AI, since nuclear medicine physicians are more likely to trust and accept a deep learning model’s output if they can see the reasons behind them and if it fits a medical logic [29].

The primary limitation of this study is the low specificity, resulting in a significant number of false positives, although this was a deliberate trade-off for higher sensitivity, especially since we used only 2 projections instead of 120. In practice, a false positive from our AI would simply undergo the full SPECT, which he was getting anyway. Future enhancements could involve a more nuanced decision scheme: for instance, if the model output is very low (clear normal) or very high (clear abnormal). In other words, the model could potentially identify both ends of the spectrum (definitely normal or definitely abnormal) early, and only ambiguous cases could require full-length scanning or re-scanning.

Furthermore, the model was trained, validated, and tested on data from a single center, which may limit its generalizability. Future studies should include multi-center data to enhance the robustness and applicability of the model across diverse populations, imaging systems, and protocols. By doing so, we would increase the number of patients in the independent testing set, thereby achieving a more accurate 95% confidence interval. Another limitation is that our gold standard for labeling ‘normal’ or ‘abnormal’ DaT-SPECT was based on routine interpretation, which may include misclassifications. However, we chose to consider doubtful exams as abnormal to maximize reliability in excluding normal SPECT. Furthermore, it is well established that visual interpretation of DaT-SPECT by senior practicians is pretty reliable for dopaminergic status, and thus a Parkinson’s or Parkinson-like disease diagnosis [30,31].

Previous studies using deep learning on DaT-SPECT have generally used the complete 3D scan data. For example, a custom CNN in 2022 achieved ~96% accuracy on classifying DAT-SPECT as normal vs Parkinson’s, with both sensitivity and specificity in the 93–99% range [7]. Those models, with a really high specificity, are designed to be diagnostic classifiers. However, they require the entirety of imaging data [32]. In contrast, our model intentionally operates with incomplete data (only the first projection), a more challenging task, in order to classify a patient with only an initial glimpse. It is unsurprising that our model’s specificity is lower; there is inherently less information in one projection and no robust semiquantitative uptake values. The fact that we still attained ~98% sensitivity is encouraging, and it suggests that the first projection carries enough signal (the relative counts in the striatal area versus the background) to detect most abnormal cases. If needed, specificity could likely be improved by incorporating more data per patient without greatly prolonging acquisition, for example using the first few projections or the first minutes could offer multiple views of the striatum and reduce false positives.

Another point of comparison is with classical quantitative analysis methods of DaT-SPECT (e.g., striatal binding ratios, caudate/putamen ratios, etc.), which are often used alongside visual reads. These quantitative metrics, when combined with machine learning algorithms like support vector machines or decision trees, have shown high accuracy in distinguishing Parkinson’s diseases from controls [33]. This suggests that much of the information the CNN is using overlaps with what is captured by ROI-based counts in the striatum. Our model did not explicitly compute any binding ratios, yet by learning from raw images it likely internalized a similar concept, such as recognizing the expected high uptake in basal ganglia for normals vs reduced uptake for abnormals.

Beyond our study, it should be noted that CNNs are not only used for classification tasks, but also for exploring deep learning to improve image quality and quantification. For example, deep generative models have been used to perform SPECT image attenuation corrections and improve quantification of striatal uptake [34]. Similarly, others have introduced attention mechanisms and novel network architectures to enhance SPECT images [35,36].

## 5. Conclusions

This study presents a CNN model capable of predicting 123I-FP-CIT SPECT results from the first acquired projection only and the age, demonstrating high sensitivity (98%) and NPV (96.3%). The potential clinical impact of this model lies in its ability to rapidly and reliably exclude presynaptic dopaminergic loss in normal DaT-SPECT, in less than a minute of acquisition, providing useful medical insights in cases where compliance with full acquisition is a concern. The methodology was oriented toward speed and efficiency, the simplicity of our preprocessing and model pipeline means it could be readily implemented in a clinical setting, even on standard computing resources, as a rule out test. Future research will be necessary to validate and refine this approach with other centers, ensuring broader applicability and improved performance.

## Figures and Tables

**Table 1:** Comparative table of state-of-the-art AI models in dopaminergic imaging. Related works using AI on dopaminergic imaging. Prior studies used either full SPECT data (reconstructed 3D images from all the projections) or PET, often with complex models, to classify Parkinson’s Disease (PD) or other conditions vs normal outputs. Our approach is novel in using merely two projections.

Study (Year)	Imaging Modality (Projections)	AI Model Type	Input Features	Output Task	Performance
Prashanth et al. (2016) [8]	DaT-SPECT (full 3D volume)	SVM (support vector machine)	Parkinson’s Progression Markers Initiative (PPMI) database: striatal shape and surface features	Normal vs. PD	Accuracy 96.1%, Se 95.7%, Sp 77.3%
Ortiz et al. (2019) [9]	DaT-SPECT (3D isosurfaces)	CNN (AlexNet/LeNet)	Isosurface-derived features	Normal vs. PD	Accuracy 95.1%, Se 95.5%, Sp 94.8%
Chien et al. (2020) [10]	DaT-SPECT (full 3D volume)	ANN (transfer learning)	Segmented putamen ROI	PD vs. other Parkinsonisms	Accuracy 86%, Se 81.8%, Sp 88.6%
Magesh et al. (2020) [11]	DaT-SPECT (2D slices from all projections)	CNN (VGG16 + LIME)	Striatal ROI in slices	Normal vs. PD	Accuracy 95.2%, Se 97.5%, Sp 90.9%
Hathaliya et al. (2022) [12]	DaT-SPECT (full 3D volume)	CNN (custom)	Parkinson’s Progression Markers Initiative (PPMI) database: striatal ROI from slices	Normal vs. PD	Accuracy 88.9%
Thakur et al. (2022) [13]	DaT-SPECT (augmented slices from 3D volumes)	CNN (DenseNet-121)	Full slices with attention	Normal vs. PD	Accuracy 99.2%, Se 99.2%, Sp 99.4%
Kurmi et al. (2022) [14]	DaT-SPECT (full 3D volume)	Ensemble of 4 CNN	Slices inputs	Normal vs. PD	Accuracy 98.4%, Se 98.8%, Sp 97.7%
Budenkotte et al. (2024) [15]	DaT-SPECT (full 3D volume)	Ensemble of 5 ResNet-style CNNs + Uncertainty-Detection Module	Full pre-processed SPECT aggregated to 12 mm axial slabs	Normal vs. PD	Accuracy 98.0%
Yoon et al. (2024) [16]	DaT-SPECT and 18F-AV133 PET (all projections)	3D-to-2D Knowledge-Distillation framework (from Teacher 3D-CN to Student 2D-CNN)	Full 3D; stacked maximum-intensity-projection and representative 2D slices	Normal vs. PD	Accuracy 98.3%

DaT-SPECT = dopamine transporter SPECT, PET = positron emission tomography with dopaminergic tracer. ROI = Region Of Interest. Se = sensitivity, Sp = specificity).

**Table 2:** DaT-SPECT’s distribution.

	Negative DaT-SPECT: Normal	Positive DaT-SPECT: Abnormal	Total
Training set	Score 0: 359	Score 1: 9 Score 2: 9 Score 3: 241	618
Validation set	Score 0: 157	Score 1: 4 Score 2: 3 Score 3: 100	264
Testing set	Score 0: 50	Score 1: 0 Score 2: 0 Score 3: 50	100
Total	566	416	982

**Table 3:** Confusion matrix.

	Actual Negative	Actual Positive	TOTAL	
Predicted Negative	**26 ** (TN)	**1** (FN)	**27**	**NPV = 96.3%** *CI 95% [81.7–99.3]*
Predicted Positive	**24** (FP)	**49** (TP)	**73**	**PPV = 67.1%** *CI 95% [55.7–76.8]*
**TOTAL**	**50**	**50**	**100**	
	**Se = 98.0%** *CI 95% [89.5–99.6]*	**Sp = 52.0%** *CI 95% [38.5–65.2]*		

TN: True Negative; FN: False Negative; TP: True Positive; FP: False Positive; Se: sensitivity; Sp: specificity; NPV: negative predictive value; PPV: positive predictive value; CI: confidence interval.

**Figure A1:**
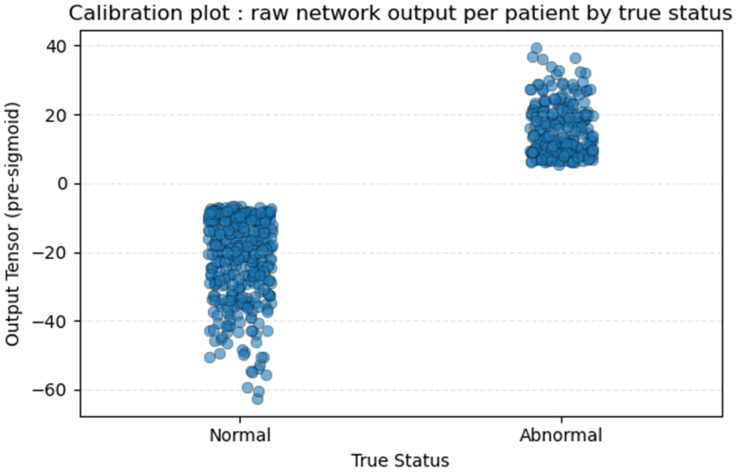
Calibration Plot of Model in Training Set.

## Data Availability

The datasets generated during the current study are not publicly available due to data protection policies, but are available from the corresponding author on reasonable request.

## Abbreviations

The following abbreviations are used in this manuscript:

AI	Artificial intelligence
CI	Confidence intervals
CNN	Convolutional Neural Network
DaT	Dopamine Transporter ligands
FN	False Negative
FP	False Positive
PCI	Pathological Confidence Index
SPECT	Single-Photon Emission Computed Tomography
ReLU	Rectified Linear Unit
Se	Sensitivity
Sp	Specificity
TN	True Negative
TP	True Positive
VGG16	Visual Geometry Group of the University of Oxford—16 layers

## Appendix A

**Table A1:** TRIPOD-AI Checklist.

Section	Topic	Item	Development/ Evaluation	Checklist Item	Page and Comments
TITLE	Title	1	D;E	Identify the study as developing or evaluating the performance of a multivariable prediction model, the target population, and the outcome to be predicted.	p1
ABSTRACT	Abstract	2	D;E	Report an abstract addressing each item in the TRIPOD+AI for Abstracts checklist.	p1
INTRODUCTION	Background	3a	D;E	Explain the healthcare context (including whether diagnostic or prognostic) and rationale for developing or evaluating the prediction model, including references to existing models.	p2–3 §1.1 and §1.2— Table 1
		3b	D;E	Describe the target population and the intended purpose of the prediction model in the care pathway, including its intended users.	p2 §1.1 and §1.3
		3c	D;E	Describe any known health inequalities between sociodemographic groups.	NA
	Objectives	4	D;E	Specify the study objectives, including whether the study describes development or validation of the prediction model (or both).	p2 §1.3
METHODS	Data	5a	D;E	Describe the sources of data separately for the development and evaluation datasets (e.g., randomised trial, cohort, routine care or registry data), the rationale for using these data, and representativeness of the data.	p4 §2.1
		5b	D;E	Specify the dates of the collected participant data, including start and end of participant accrual; and, if applicable, end of follow-up.	p4 §2.1
	Participants	6a	D;E	Specify key elements of the study setting (e.g., primary care, secondary care, general population) including the number and location of centres.	p4 §2.1
		6b	D;E	Describe the eligibility criteria for study participants.	p4 §2.1
		6c	D;E	Give details of any treatments received, and how they were handled during model development or evaluation, if relevant.	NA— diagnostic imaging
	Data preparation	7	D;E	Describe any data pre-processing and quality checking, including whether this was similar across relevant sociodemographic groups.	p5–6 §2.2
	Outcome	8a	D;E	Clearly define the outcome that is being predicted and the time horizon, including how and when assessed, the rationale for choosing this outcome, and whether the method of outcome assessment is consistent across sociodemographic groups.	p4 §2.1
		8b	D;E	If outcome assessment requires subjective interpretation, describe the qualifications and demographic characteristics of the outcome assessors.	p4 §2.1
		8c	D;E	Report any actions to blind assessment of the outcome to be predicted.	p4 §2.1
	Predictors	9a	D	Describe the choice of initial predictors (e.g., literature, previous models, all available predictors) and any pre-selection of predictors before model building.	p2 §1.2 and Table 1
		9b	D;E	Clearly define all predictors, including how and when they were measured (and any actions to blind assessment of predictors for the outcome and other predictors).	p5–7 §2.2 and §2.3
		9c	D;E	If predictor measurement requires subjective interpretation, describe the qualifications and demographic characteristics of the predictor assessors.	NA
	Sample size	10	D;E	Explain how the study size was arrived at (separately for development and evaluation), and justify that the study size was sufficient to answer the research question. Include details of any sample size calculation.	p4 §2.1
	Missing data	11	D;E	Describe how missing data were handled. Provide reasons for omitting any data.	p4 §2.1
	Analytical methods	12a	D	Describe how the data were used (e.g., for development and evaluation of model performance) in the analysis, including whether the data were partitioned, considering any sample size requirements.	p5–6 §2.2
		12b	D	Depending on the type of model, describe how predictors were handled in the analyses (functional form, rescaling, transformation, or any standardisation).	p5–7 §2.2 and §2.3
		12c	D	Specify the type of model, rationale2, all model-building steps, including any hyperparameter tuning, and method for internal validation.	p5–7 §2.2 and §2.3—Figure 2
		12d	D;E	Describe if and how any heterogeneity in estimates of model parameter values and model performance was handled and quantified across clusters (e.g., hospitals, countries). See TRIPOD-Cluster for additional considerations	NA— single-centre study
		12e	D;E	Specify all measures and plots used (and their rationale) to evaluate model performance (e.g., discrimination, calibration, clinical utility) and, if relevant, to compare multiple models.	p6–7 §2.3; p8 §3.2—Figure 3 and Figure A1 calibration plot
		12f	E	Describe any model updating (e.g., recalibration) arising from the model evaluation, either overall or for particular sociodemographic groups or settings.	NA
		12g	E	For model evaluation, describe how the model predictions were calculated (e.g., formula, code, object, application programming interface).	p5–6 §2.2
	Class imbalance	13	D;E	If class imbalance methods were used, state why and how this was done, and any subsequent methods to recalibrate the model or the model predictions.	NA
	Fairness	14	D;E	Describe any approaches that were used to address model fairness and their rationale.	NA
	Model output	15	D	Specify the output of the prediction model (e.g., probabilities, classification). Provide details and rationale for any classification and how the thresholds were identified.	p6–7 §2.3
	Development versus evaluation	16	D;E	Identify any differences between the development and evaluation data in healthcare setting, eligibility criteria, outcome, and predictors.	p4 §2.1
	Ethical approval	17	D;E	Name the institutional research board or ethics committee that approved the study and describe the participant-informed consent or the ethics committee waiver of informed consent.	p13
OPEN SCIENCE	Funding	18a	D;E	Give the source of funding and the role of the funders for the present study.	p13
	Conflicts of interest	18b	D;E	Declare any conflicts of interest and financial disclosures for all authors.	p13
	Protocol	18c	D;E	Indicate where the study protocol can be accessed or state that a protocol was not prepared.	p13
	Registration	18d	D;E	Provide registration information for the study, including register name and registration number, or state that the study was not registered.	p1
	Data sharing	18e	D;E	Provide details of the availability of the study data.	p13
	Code sharing	18f	D;E	Provide details of the availability of the analytical code.	p13
PATIENT AND PUBLIC INVOLVEMENT	Patient and public involvement	19	D;E	Provide details of any patient and public involvement during the design, conduct, reporting, interpretation, or dissemination of the study or state no involvement.	p4 and p13
RESULTS	Participants	20a	D;E	Describe the flow of participants through the study, including the number of participants with and without the outcome and, if applicable, a summary of the follow-up time.	p7 §3.1– Table 2
		20b	D;E	Report the characteristics overall and, where applicable, for each data source or setting, including the key dates, key predictors (including demographics), treatments received, sample size, number of outcome events, follow-up time, and amount of missing data. A table may be helpful. Report any differences across key demographic groups.	p7 §3.1– Table 2
		20c	E	For model evaluation, show a comparison with the development data of the distribution of important predictors (demographics, predictors, and outcome).	NA—no other model predicting from only 2 projections
	Model development	21	D;E	Specify the number of participants and outcome events in each analysis (e.g., for model development, hyperparameter tuning, model evaluation).	p7
	Model specification	22	D	Provide details of the full prediction model (e.g., formula, code, object, API) to allow predictions in new individuals and to enable third-party evaluation and implementation, including any restrictions to access or re-use (e.g., freely available, proprietary).	p6—Figure 2
	Model performance	23a	D;E	Report model performance estimates with confidence intervals, including for any key subgroups (e.g., sociodemographic).	p8–9 §3.2 and §3.3—Table 3
		23b	D;E	If examined, report results of any heterogeneity in model performance across clusters. See TRIPOD Cluster for additional details.	NA
	Model updating	24	E	Report the results from any model updating, including the updated model and subsequent performance.	NA
DISCUSSION	Interpretation	25	D;E	Give an overall interpretation of the main results, including issues of fairness in the context of the objectives and previous studies.	p11–12
	Limitations	26	D;E	Discuss any limitations of the study (such as a non-representative sample, sample size, overfitting, missing data) and their effects on any biases, statistical uncertainty, and generalizability.	p11–12
	Usability of the model in the context of current care	27a	D	Describe how poor quality or unavailable input data (e.g., predictor values) should be assessed and handled when implementing the prediction model.	p12
		27b	D	Discuss whether users will be required to interact in the handling of the input data or use of the model, and what level of expertise is required of users.	p11
		27c	D;E	Discuss any next steps for future research, with a specific view to applicability and generalizability of the model.	p12

Note: pX = page X of manuscript. NA = not applicable to this study.
